# Supplementary material for: High-throughput screening and validation of antibodies against synaptic proteins to explore opioid signaling dynamics
Source: Commun Biol. 2021 Feb 22;4:238. doi: 10.1038/s42003-021-01744-8 (PMC7900253; doi:10.1038/s42003-021-01744-8)
Supplement: Supplementary file 5 — Supplementary Information [file 42003_2021_1744_MOESM5_ESM.pdf]

Supplementay Figure 1

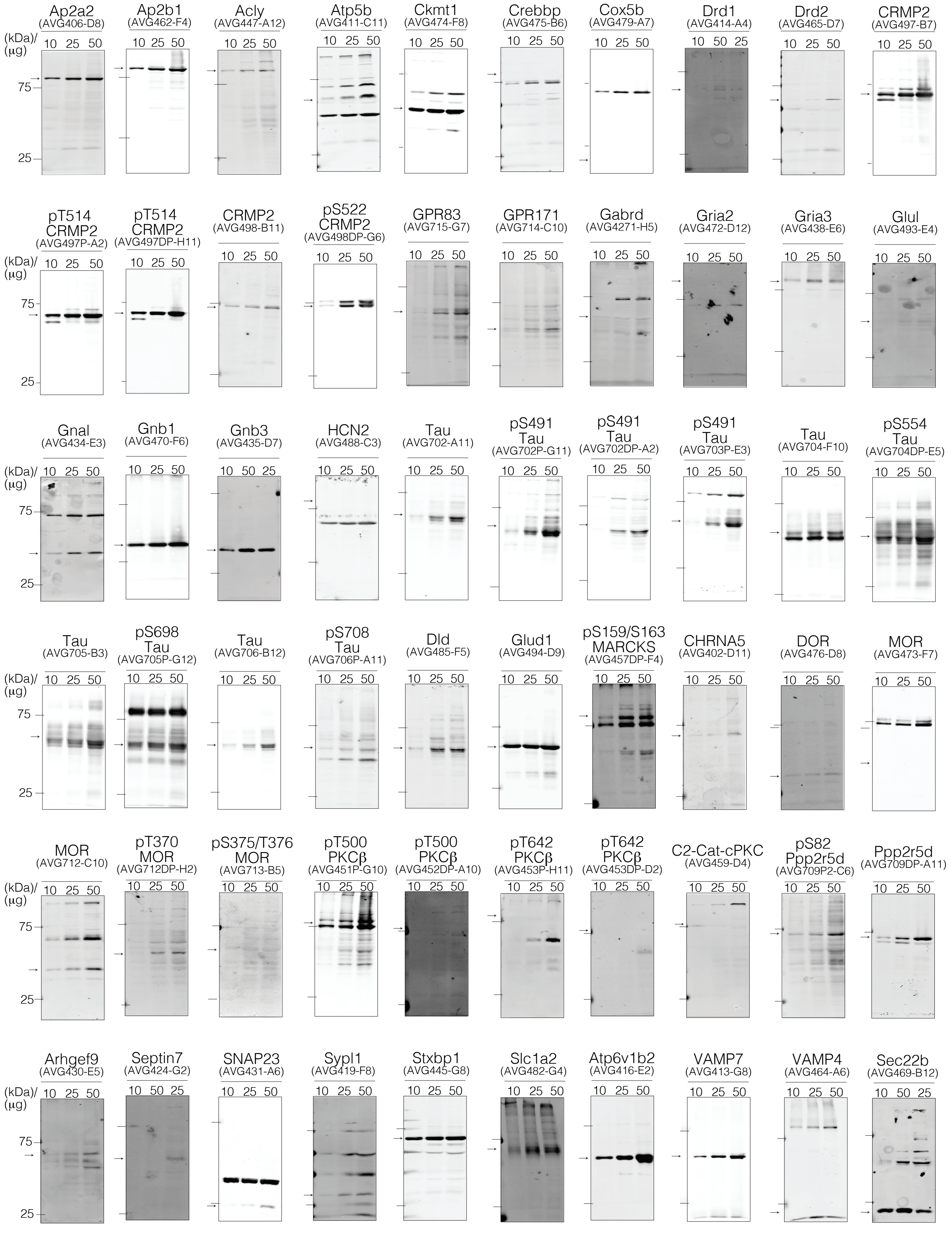

Supplementary Fig. 1: Antibody validation of synaptic proteins using denaturating extraction buffer. Western Blot analysis using total brain protein extract at different protein concentrations. The arrow, in each blot, indicates the predicted protein molecular weight.

Supplementay Figure 2

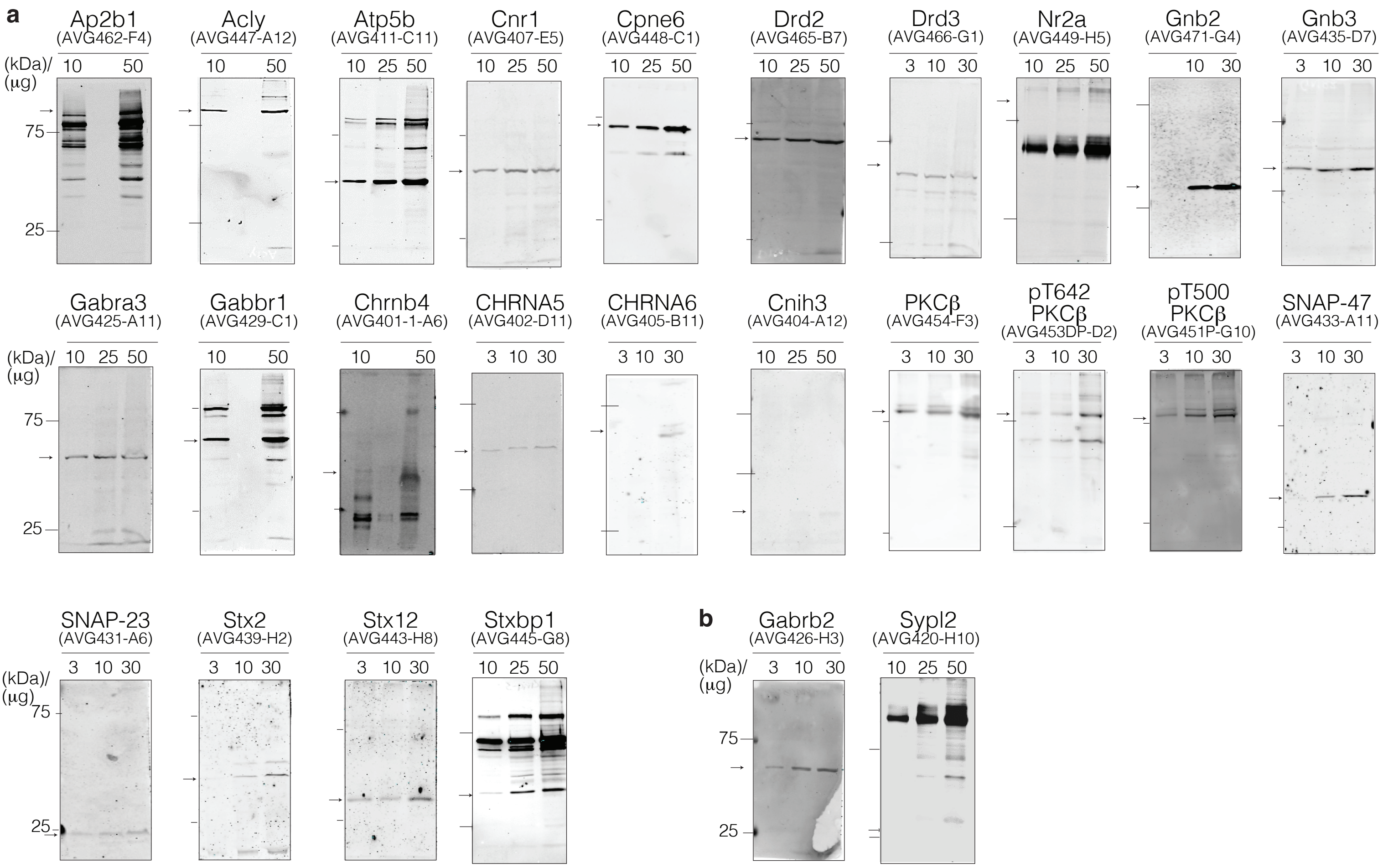

Supplementary Fig. 2: Antibody validation of synaptic proteins using non-denaturating extraction buffer. Western Blot analysis using total brain protein extract (a), and synaptosomal protein fractions (b) at different protein concentrations. The arrow in each blot, indicates the predicted protein molecular weight.

Supplementay Figure 3

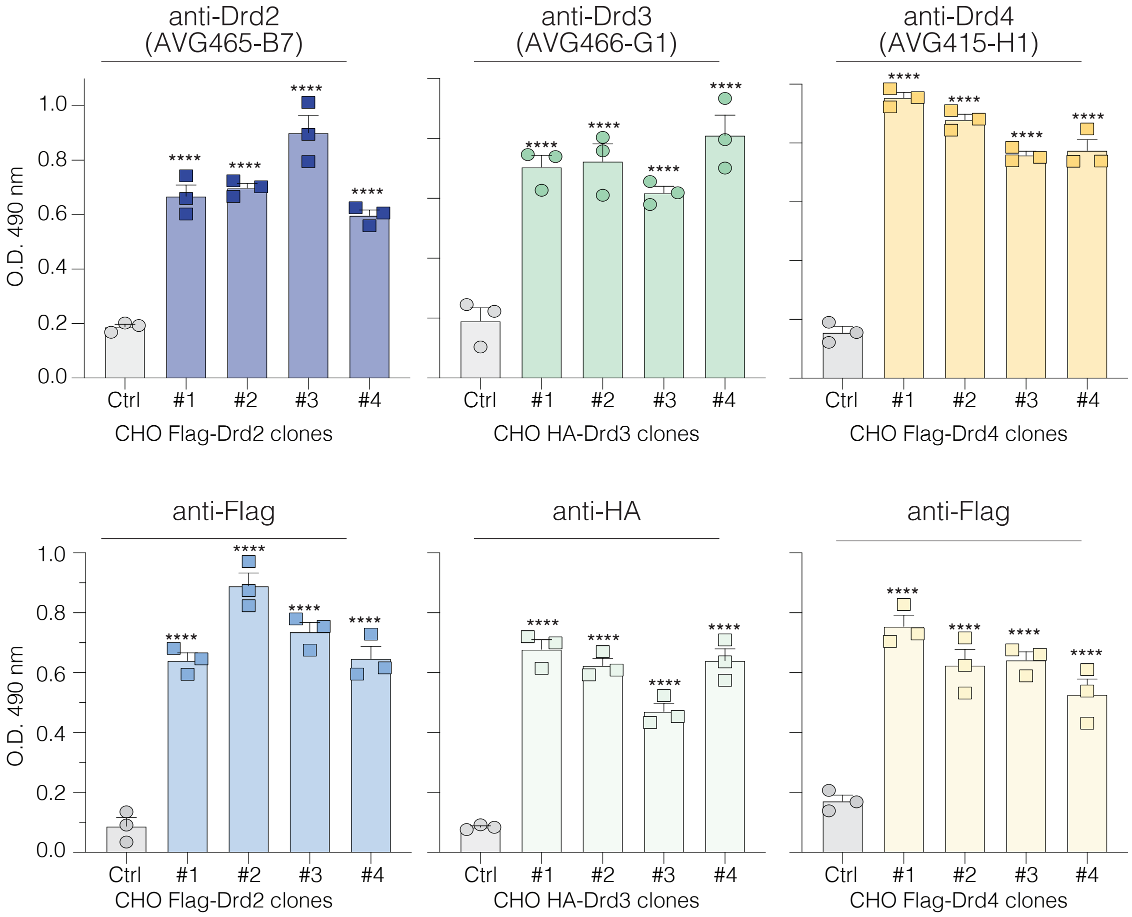

Supplementary Fig. 3: Expression of tagged protein for antibody validation. ELISA with CHO cells and different clones of CHO cells overexpressing N-terminally tagged dopamine receptor. The Drd2 (dopamine D2 receptor) and Drd4 (dopamine D4 receptor) plasmids have a Flag-tag at the N-terminus, and the Drd3 (dopamine D3 receptor) plasmid has a HA-tag at the N-terminus. ELISA was performed using antibodies specific for each receptor (top panel) and for the respective tag (bottom panel). Data are mean ± SE of three experiments, P value < 0.0001; One-way ANOVA.

Supplementay Figure 4

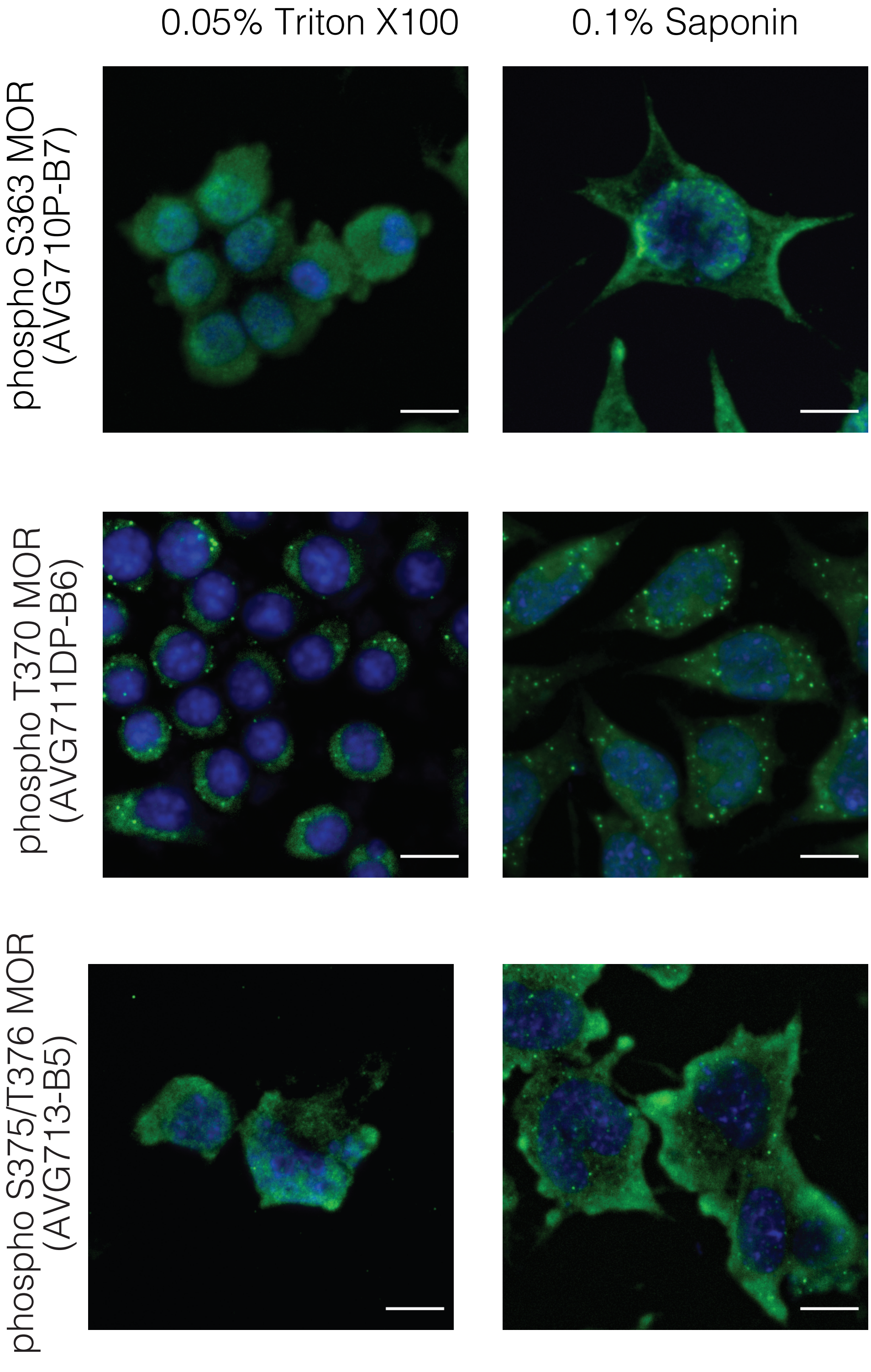

Supplementary Fig. 4: Effects of detergents on sub-cellular protein distribution detected by immunofluorescence. Immunofluorescence analysis was carried out in Neuro2A cells. Representative images of cells stained with phosphospecific MOR antibodies using two different protocols: fixation with 4% PFA and cell permeabilization using Triton-X-100 (0.05%) (left panels); fixation with 4% PFA followed by 100% methanol, and permeabilization using 0.1% saponin (right panels). Cells were acquired using the InCell microscope (40x).

Supplementay Figure 5

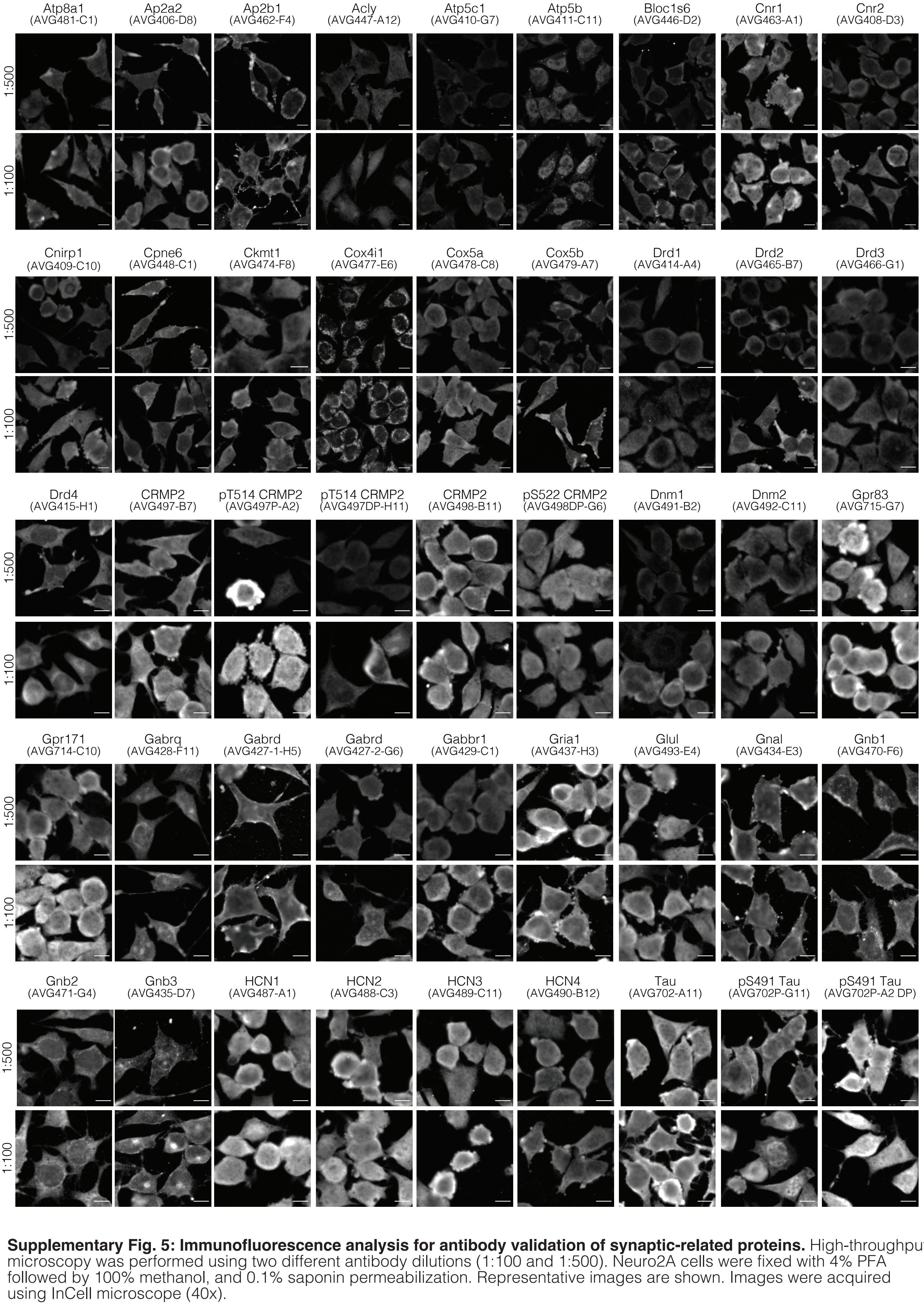

Supplementary Fig. 5: Immunofluorescence analysis for antibody validation of synaptic-related proteins. High-throughput microscopy was performed using two different antibody dilutions (1:100 and 1:500). Neuro2A cells were fixed with 4% PFA followed by 100% methanol, and 0.1% saponin permeabilization. Representative images are shown. Images were acquired using InCell microscope (40x).

Supplementay Figure 6

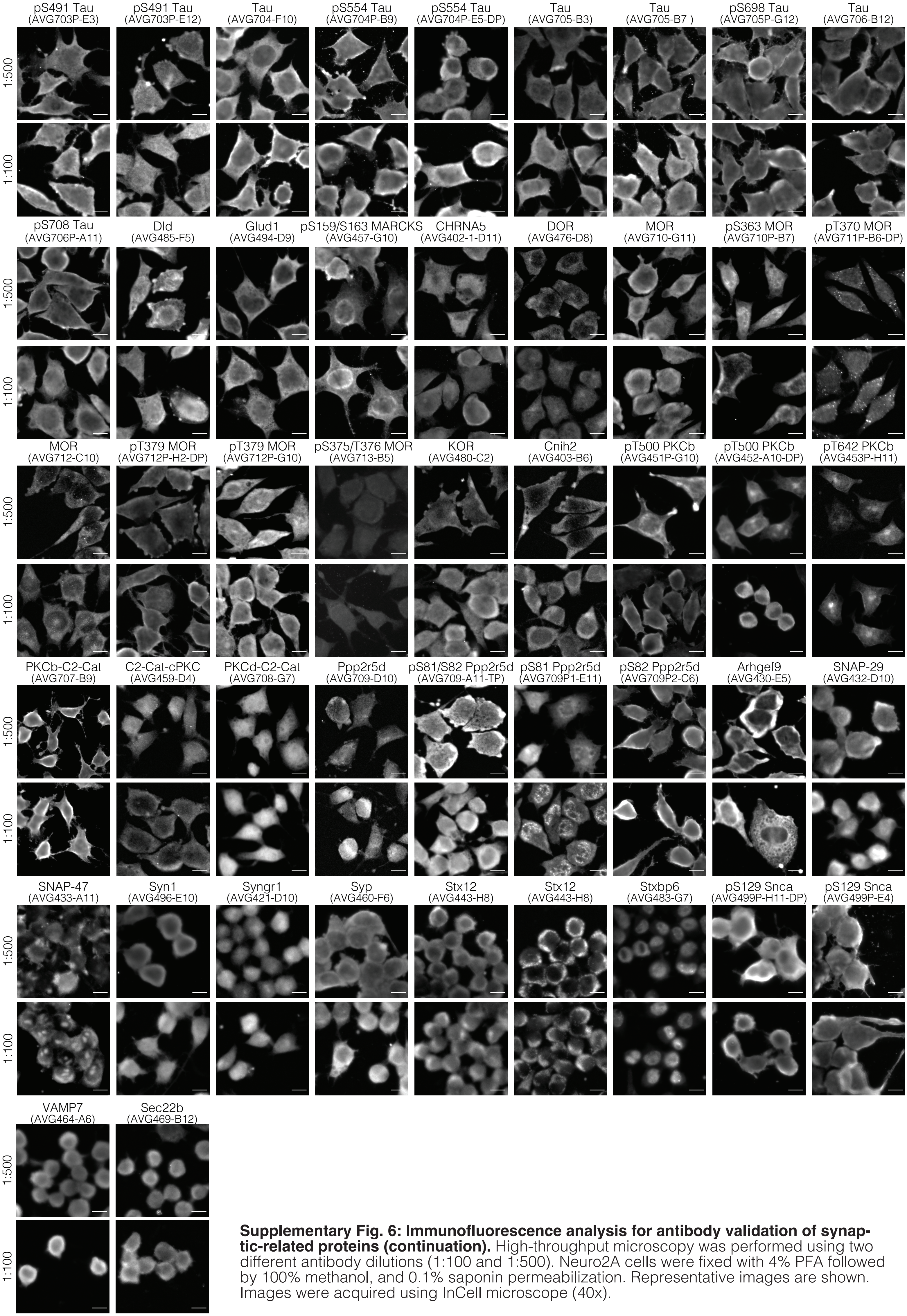

Supplementary Fig. 6: Immunofluorescence analysis for antibody validation of synaptic-related proteins (continuation). High-throughput microscopy was performed using two different antibody dilutions (1:100 and 1:500). Neuro2A cells were fixed with 4% PFA followed by 100% methanol, and 0.1% saponin permeabilization. Representative images are shown. Images were acquired using InCell microscope (40x).

Supplementay Figure 7

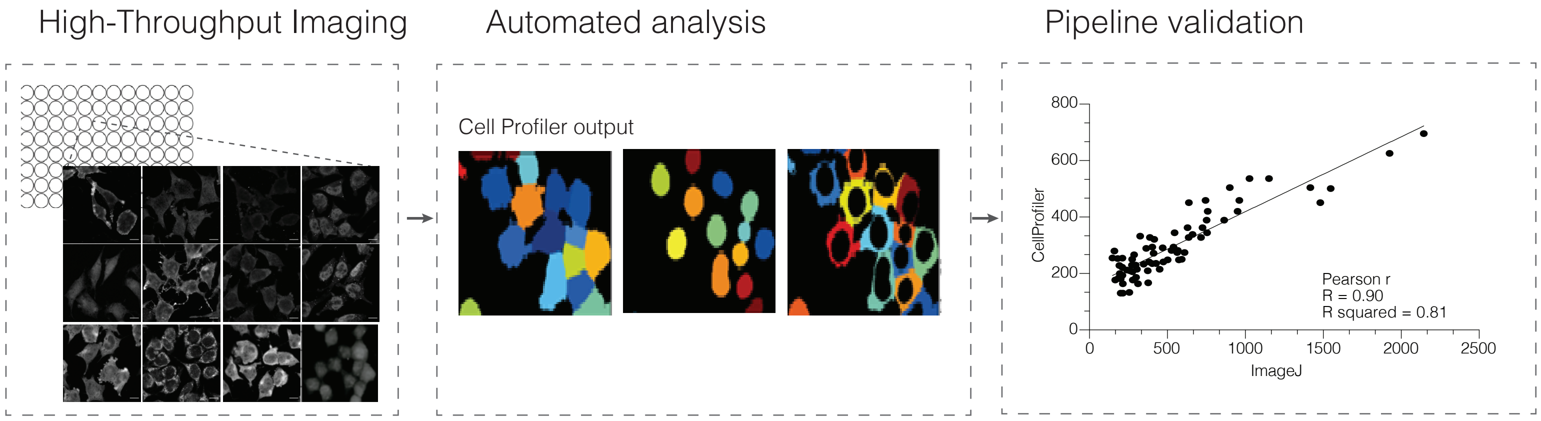

Supplementary Fig. 7: High-throughput microscopy as a strategy to validate rAbs. Overview of the HTM pipeline. Using a multiwell format it is possible to test multiple antibodies in different cell lines. High-throughput imaging (left panel) and automated image analysis (middle panel) are used to extract multiparametric staining features. Images were acquired using the InCell microscope (GE Healthcare). Representative images as result of the Cell Profiler analysis are shown (middle panel). Correlation graph between data obtained from Cell Profiler and ImageJ (right panel) is shown. Linear regression, R square 0.81 **** p value < 0.0001.

Supplementay Figure 8

a

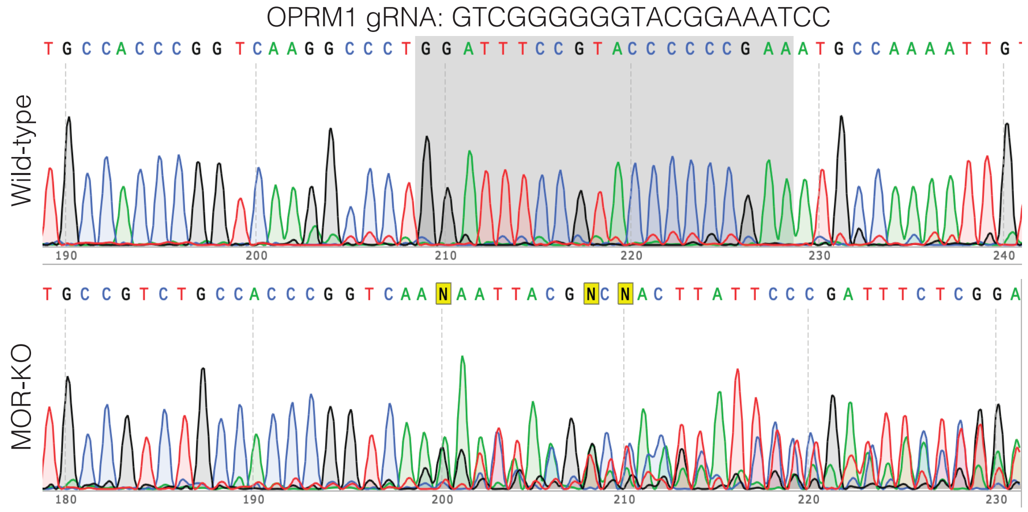

b

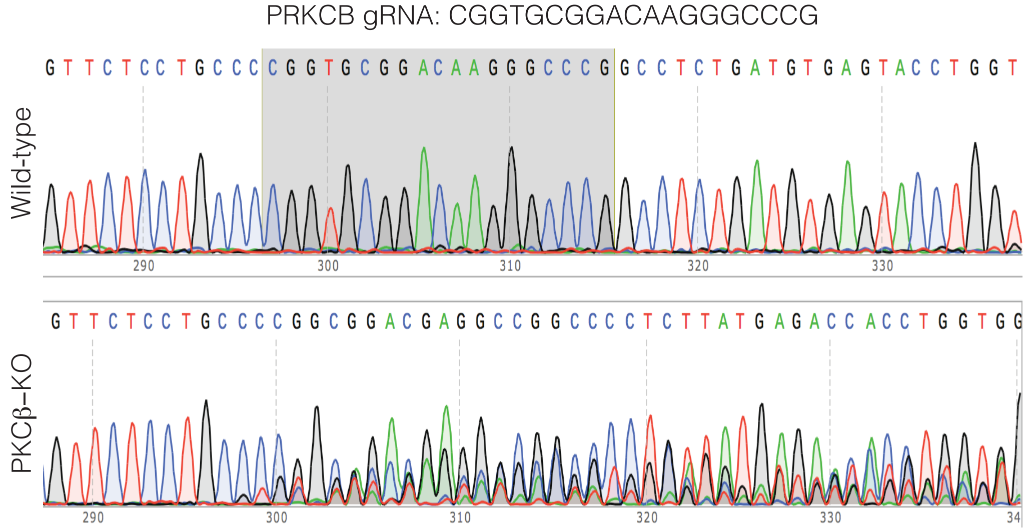

c

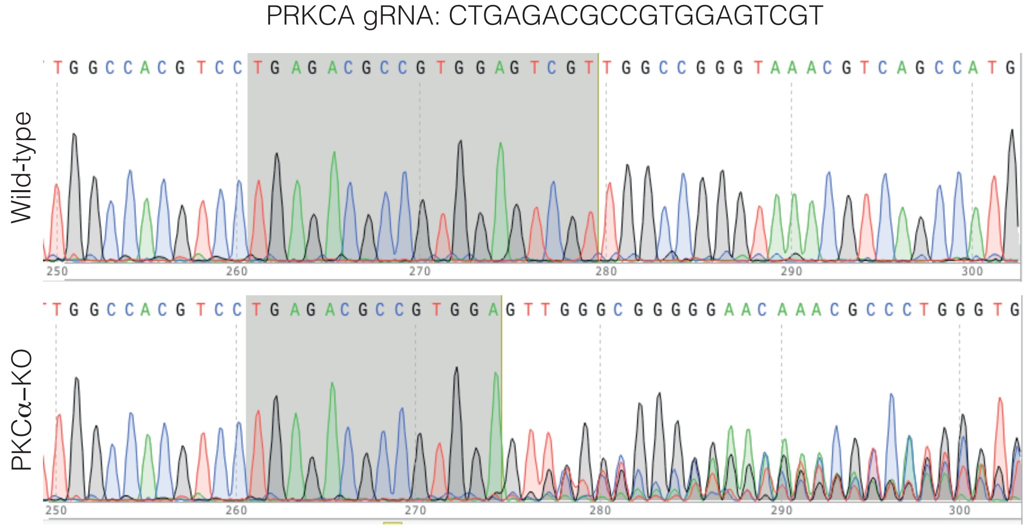

Supplementary Fig. 8: Generation of Neuro2A MOR-KO, Neuro2A PKCβ-KO or Neuro2A PKCα-KO cell lines using genome editing. DNA sequence analysis of PCR amplified genomic DNA in Neuro2A cells. MOR (a), PKCβ (b) or PKCα (c) were knocked out from Neuro2A cells, using CRISPR/Cas9 strategy. The gRNAs were designed (highlighted in gray) using the webtool CCTop (https://crispr.cos.uni-heidelberg.de) and cloned into the PX459 V2.0 plasmid. Neuro2A cells were transfected with these constructs using Turbofectamine. After 24 hours, cells were selected using puromycin (1 μg/mL) for 48 hours. The DNA was extracted using the Qiagen kit, the genomic PCR was performed using Q5-high fidelity polymerase and the amplified DNA subjected to DNA sequencing.

Supplementay Figure 9

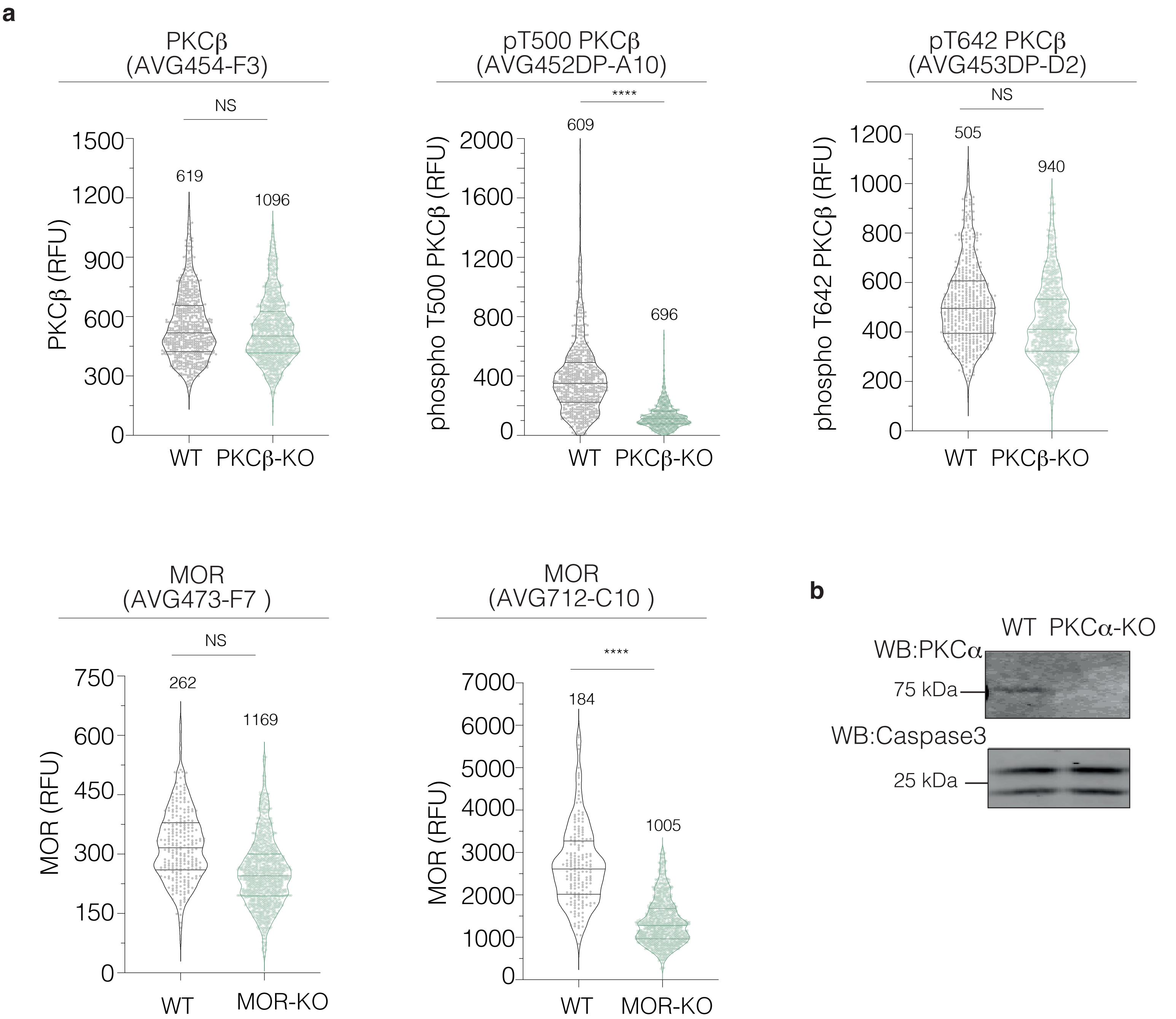

Supplementary Fig. 9: Neuro2A MOR-KO, Neuro2A PKCβ-KO or Neuro2A PKCα-KO cell line validation. Neuro2A, Neuro2A PKCβ-KO, Neuro2A MOR-KO and Neuro2A PKCα-KO cells were probed with antibodies against PKCβ, MOR and PKCα, respectively. a Images were acquired using the InCell microscope (40x) and fluorescence intensity measured by Cell Profiler software. Volcano plot, each dot represented one cell. Data are mean ± SD of two experiments, number of counted cells are indicated. Data acquired from 20 fields. **** p value < 0.0001; t test, for the immunostaining using anti-phosphoT500 PKCβ (AVG452DP-A10), and anti-MOR (AVG712-C10). Non-significant; t test, for the immunostaining using anti-PKCβ (AVG454-F3), anti-phospho T642 PKCβ (AVG453DP-D2), and anti-MOR (AVG473-F7). b Western Blot analysis using total protein extract from Neuro2A and Neuro2A PKCα-KO cells. The blots were probed with anti-PKCα antibody (Santa Cruz; sc-8393) and anti-Caspase3 (Cell Signaling, #9662), as endogenous control.

Supplementay Figure 10

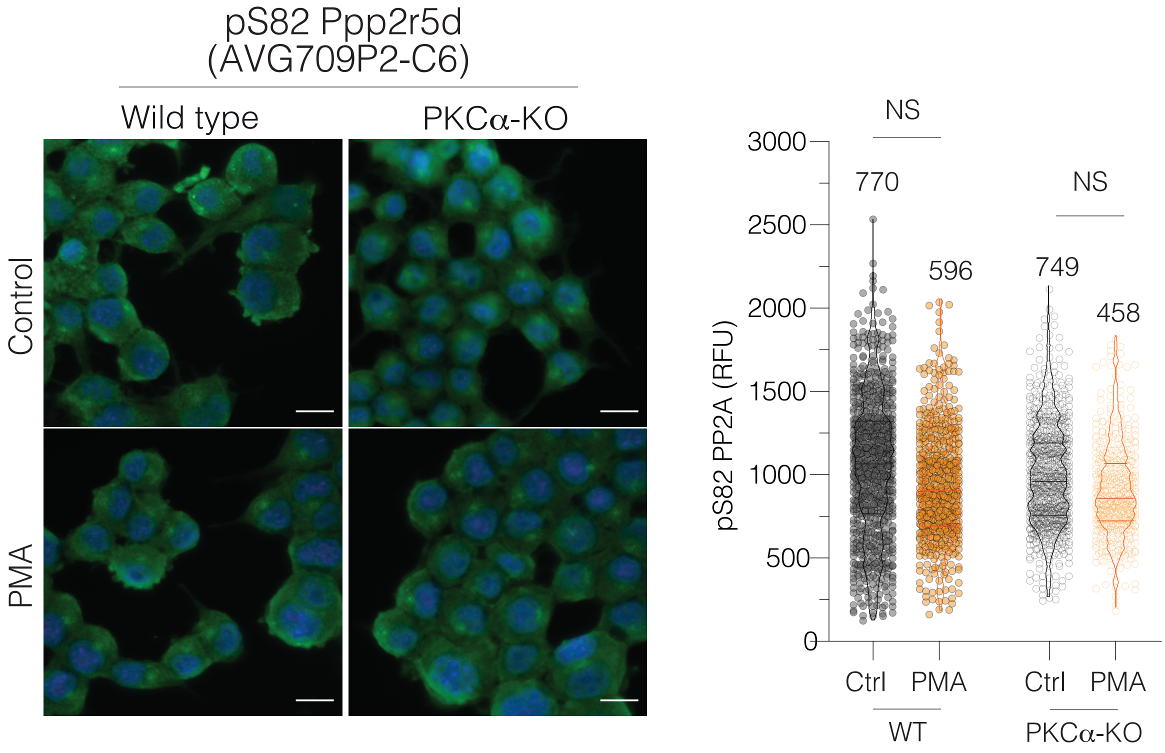

Supplementary Fig. 10: Immunofluorescence analysis for phospho Ser82 Ppp2r5d (AVG709P2-C6). Neuro2A and Neuro2A PKCα-KO cells were treated with PMA (100 nM) for 30 minutes. Cells were stained with DAPI (blue) and antibodies (green). Images were acquired using InCell microscope (GE Healthcare). Fluorescence intensity was measured using Cell Profiler software. NS; non-significant; t test. Data are mean ± SD of two experiments. RFU indicates the integrated fluorescence intensity units.

Supplementary Table 1: Relative mRNA and Protein expression in Neuro2A and HEK293 cell lines

		ELISA (O.D.)						RNA (RANK)								Relative expression									
Clone ID	Antigen Gene	N2A			HEK293T			Neuro2A				HEK				N2A_PTN			HEK_PTN			N2A_RNA			
AVG447	Acly	0.42	0.47	0.42	0.18	0.20	0.18	96	96	96	96	89	89	91	90	0.67	0.75	0.68	0.29	0.33	0.3	0.52	0.52	0.52	0.52
AVG446	Bloc1s6	0.38	0.35	0.25	0.14	-	0.16	80	81	81	82	85	86	86	86	0.8	0.75	0.52	0.29	0.26	0.34	0.48	0.49	0.49	0.49
AVG457	Marcks	0.27	0.19	0.19	0.12	0.12	0.06	88	88	89	88	87	87	86	88	0.88	0.6	0.6	0.4	0.38	0.2	0.5	0.5	0.51	0.5
AVG423	Septin 6	0.54	0.54	0.65	0.09	0.13	0.09	95	94	94	96	77	78	76	78	0.79	0.8	0.96	0.14	0.19	0.13	0.55	0.55	0.55	0.56
AVG445	Stxbp1	0.23	0.26	0.36	0.12	0.07	0.06	94	94	94	95	83	84	83	84	0.62	0.71	0.97	0.31	0.18	0.15	0.53	0.53	0.53	0.53
AVG420	Sypl2	0.20	0.32	0.42	0.23	0.20	0.22	28	22	34	30	47	48	47	47	0.38	0.6	0.79	0.43	0.38	0.42	0.37	0.29	0.45	0.4
AVG439	Stx2	0.57	0.57	0.63	0.30	0.29	0.22	88	88	88	88	81	81	81	81	0.66	0.66	0.74	0.35	0.34	0.26	0.52	0.52	0.52	0.52
AVG443	Stx12	0.70	0.53	0.51	0.33	0.25	0.27	90	90	90	90	91	91	90	90	0.8	0.62	0.59	0.38	0.29	0.32	0.5	0.5	0.5	0.5
AVG416	Atp6v1b2	0.64	0.75	0.61	0.32	0.24	0.23	98	99	99	99	92	92	93	92	0.69	0.81	0.65	0.34	0.26	0.25	0.51	0.52	0.52	0.52
AVG412	VAMP3	0.69	0.53	0.51	0.30	0.22	0.25	92	92	92	93	91	92	91	92	0.83	0.64	0.61	0.36	0.27	0.3	0.5	0.5	0.5	0.51
AVG413	VAMP7	1.74	1.61	1.68	1.68	1.61	1.69	91	91	91	91	93	93	93	93	0.52	0.48	0.5	0.5	0.48	0.51	0.49	0.49	0.49	0.49
AVG469	Sec22b	0.21	0.18	0.17	0.01	0.00	0.01	87	87	88	90	90	91	91	91	1.09	0.94	0.86	0.06	0.01	0.06	0.49	0.49	0.49	0.5
AVG421	Syngr1	0.57	0.56	0.63	0.28	0.27	0.20	82	83	83	85	65	65	64	64	0.68	0.67	0.75	0.33	0.32	0.24	0.55	0.56	0.56	0.58
AVG460	Syp	1.00	0.81	0.80	0.66	0.58	0.42	79	81	80	80	51	47	44	43	0.7	0.57	0.56	0.47	0.41	0.3	0.63	0.64	0.63	0.63
AVG419	Sypl1	0.95	0.97	0.94	0.08	0.58	0.50	80	79	79	82	87	87	87	87	0.71	0.72	0.7	0.06	0.43	0.37	0.48	0.47	0.47	0.49
AVG411	ATP5B	0.44	0.41	0.52	0.07	0.05	0.11	99	99	99	99	99	99	99	99	0.82	0.76	0.98	0.14	0.09	0.21	0.5	0.5	0.5	0.5
AVG477	Cox4i1	0.21	0.24	0.21	0.09	0.09	0.03	94	94	93	94	85	85	85	85	0.73	0.82	0.73	0.31	0.31	0.12	0.53	0.53	0.52	0.53
AVG479	Cox5b	0.39	0.39	0.32	0.14	0.17	0.13	98	98	97	97	71	71	65	67	0.76	0.76	0.62	0.27	0.33	0.26	0.59	0.59	0.58	0.58
AVG475	Crebbp	0.41	0.21	0.37	0.08	0.14	0.08	92	92	91	91	87	88	89	88	0.95	0.49	0.86	0.19	0.32	0.2	0.51	0.51	0.51	0.51
AVG462	Ap2b1	1.33	1.16	1.13	0.65	0.69	0.89	95	95	95	95	96	95	96	95	0.68	0.59	0.58	0.34	0.35	0.46	0.5	0.5	0.5	0.5
AVG448	Cpne6	0.79	0.82	1.01	0.41	0.37	0.43	43	41	42	47	41	39	35	37	0.62	0.64	0.79	0.32	0.29	0.34	0.53	0.5	0.52	0.58
AVG465	Drd2	0.21	0.20	0.18	0.34	0.19	0.20	39	39	36	36	55	54	53	60	0.48	0.45	0.41	0.76*	0.42	0.46	0.42	0.42	0.39	0.39
AVG466	Drd3	0.18	0.29	0.21	0.18	0.18	0.15	18	13	12	17	30	31	28	29	0.45	0.73	0.54	0.45	0.46	0.38	0.4	0.29	0.27	0.38
AVG426	Gabrb2	0.09	0.29	0.19	0.11	0.16	0.13	39	40	42	45	22	22	19	19	0.29	0.89	0.58	0.34	0.49	0.41	0.63	0.65	0.68	0.73
AVG429	Gabbr1	0.44	0.57	0.50	0.31	0.29	0.30	79	78	79	82	72	69	72	71	0.54	0.71	0.62	0.38	0.36	0.37	0.52	0.52	0.52	0.54
AVG437	Gria1	0.41	0.48	0.42	0.46	0.33	0.24	16	13	6	12	26	30	28	29	0.52	0.62	0.54	0.6*	0.43	0.31	0.4	0.33	0.15	0.3
AVG470	Gnb1	0.62	0.65	0.74	0.54	0.47	0.34	99	99	99	99	92	91	91	91	0.55	0.58	0.66	0.49	0.42	0.3	0.52	0.52	0.52	0.52
AVG471	Gnb2	0.25	0.41	0.44	0.36	0.24	0.22	94	94	94	95	66	66	65	64	0.4	0.64	0.69	0.56	0.37	0.34	0.59	0.59	0.59	0.6
AVG435	GNB3	0.41	0.37	0.35	0.23	0.26	0.16	74	74	73	76	47	47	41	40	0.7	0.62	0.58	0.39	0.43	0.27	0.63	0.63	0.62	0.64
AVG401	CHRNB4	0.71	1.00	0.88	0.31	0.14	0.21	53	52	53	54	46	52	54	49	0.66	0.92	0.81	0.29	0.13	0.19	0.51	0.5	0.51	0.52
AVG476	Oprd1	0.23	0.33	0.33	0.24	0.24	0.21	53	55	55	56	55	55	49	57	0.44	0.63	0.62	0.45	0.45	0.39	0.49	0.51	0.51	0.51
AVG473	Oprm1	0.44	0.47	0.37	0.28	0.21	0.32	21	14	20	23	26	21	28	23	0.63	0.68	0.53	0.4	0.3	0.47	0.48	0.32	0.45	0.52
AVG431	SNAP-23	0.60	0.58	0.49	0.30	0.27	0.34	91	91	91	90	86	86	87	88	0.7	0.68	0.57	0.35	0.31	0.39	0.51	0.51	0.51	0.51
AVG432	SNAP-29	1.07	0.66	1.14	0.52	0.43	0.52	79	80	80	80	88	89	89	89	0.74	0.46	0.79	0.36	0.3	0.36	0.47	0.47	0.47	0.47

Relative expression values is the mean of the RAW measurement for each cell line divided by the sum of the Neuro2A and HEK cells RAW measurement. (RANK for mRNA, O.D for protein).
