# Supplementary material for: High-throughput screening and validation of antibodies against synaptic proteins to explore opioid signaling dynamics
Source: Commun Biol. 2021 Feb 22;4:238. doi: 10.1038/s42003-021-01744-8 (PMC7900253; doi:10.1038/s42003-021-01744-8)
Supplement: Supplementary file 6 — Description of Additional Supplementary Files [file 42003_2021_1744_MOESM6_ESM.pdf]

Description of Additional Supplementary Files

File name: Supplementary Data 1

Description: Source data for the main figures

File name: Supplementary Data 2

Description: Source data for the supplementary figures

File name: Supplementary Data 3

Description: Recombinant antibodies

File name: Supplementary Data 4

Description: High throughput microscopy and machine learning to validate rAbs.
