# Supplementary material for: High-throughput screening and validation of antibodies against synaptic proteins to explore opioid signaling dynamics
Source: Commun Biol. 2021 Feb 22;4:238. doi: 10.1038/s42003-021-01744-8 (PMC7900253; doi:10.1038/s42003-021-01744-8)
Supplement: Supplementary file 8 — Reporting Summary [file 42003_2021_1744_MOESM8_ESM.pdf]

nature research

Corresponding author(s): DBPR, COMMSBIO-19-1679-B

Last updated by author(s): November 3, 2020

Reporting Summary

Nature Research wishes to improve the reproducibility of the work that we publish. This form provides structure for consistency and transparency in reporting. For further information on Nature Research policies, see Authors & Referees and the Editorial Policy Checklist .

Statistics

For all statistical analyses, confirm that the following items are present in the figure legend, table legend, main text, or Methods section.

n/a	Confirmed	
☐	☒	The exact sample size (*n*) for each experimental group/condition, given as a discrete number and unit of measurement
☐	☒	A statement on whether measurements were taken from distinct samples or whether the same sample was measured repeatedly
☐	☒	The statistical test(s) used AND whether they are one- or two-sided<br>*Only common tests should be described solely by name; describe more complex techniques in the Methods section.*
☐	☒	A description of all covariates tested
☒	☐	A description of any assumptions or corrections, such as tests of normality and adjustment for multiple comparisons
☐	☒	A full description of the statistical parameters including central tendency (e.g. means) or other basic estimates (e.g. regression coefficient) AND variation (e.g. standard deviation) or associated estimates of uncertainty (e.g. confidence intervals)
☐	☒	For null hypothesis testing, the test statistic (e.g. *F*, *t*, *r*) with confidence intervals, effect sizes, degrees of freedom and *P* value noted<br>*Give P values as exact values whenever suitable.*
☒	☐	For Bayesian analysis, information on the choice of priors and Markov chain Monte Carlo settings
☒	☐	For hierarchical and complex designs, identification of the appropriate level for tests and full reporting of outcomes
☒	☐	Estimates of effect sizes (e.g. Cohen's *d*, Pearson's *r*), indicating how they were calculated

Our web collection on statistics for biologists contains articles on many of the points above.

Software and code

Policy information about availability of computer code

Data collection	InCell microscope (GEHealthcare); FilterMax F5 (Molecular Devices); FACSAria II (BD Biosciences).
Data analysis	Data analysis was performed using CellProfiler (Broad Institute), GraphPad Prism, Excel 2016 (Microsoft Software), ImageJ (NIH), Odyssey Imaging Systems (LI-COR Biosciences); BD FACSDiva V6.1.3 (BD Biosciences).

For manuscripts utilizing custom algorithms or software that are central to the research but not yet described in published literature, software must be made available to editors/reviewers. We strongly encourage code deposition in a community repository (e.g. GitHub). See the Nature Research guidelines for submitting code & software for further information.

Data

Policy information about availability of data

All manuscripts must include a data availability statement. This statement should provide the following information, where applicable:

- Accession codes, unique identifiers, or web links for publicly available datasets
- A list of figures that have associated raw data
- A description of any restrictions on data availability

The data that support the findings of this study are available from the corresponding author upon reasonable request.

Field-specific reporting

Please select the one below that is the best fit for your research. If you are not sure, read the appropriate sections before making your selection.

☒ Life sciences ☐ Behavioural & social sciences ☐ Ecological, evolutionary & environmental sciences

Life sciences study design

All studies must disclose on these points even when the disclosure is negative.

Sample size	No statistical methods were used to predetermine sample size. At the high-throughput microscopy analysis, 1000 - 10000 cells were acquired in each biological replicate, allowing data reproducibility.
Data exclusions	No data were excluded from the analysis.
Replication	All attempts at replication were successful, the experimental replicates were performed in triplicates/quaduplicates, in each biological replicate experiment. For the high-throughput microscopy analysis, were acquired at least 10 fields for each condition in each biological replicate. Total 2-4 biological replicates on these experiments.
Randomization	At the high-throughput microscopy analysis, the fields were radon allocated by the software.
Blinding	The investigators were blinded to group allocation during data collection and/or analysis.

Reporting for specific materials, systems and methods

We require information from authors about some types of materials, experimental systems and methods used in many studies. Here, indicate whether each material, system or method listed is relevant to your study. If you are not sure if a list item applies to your research, read the appropriate section before selecting a response.

Materials & experimental systems

n/a	Involved in the study	
☐	☒	Antibodies
☐	☒	Eukaryotic cell lines
☒	☐	Palaeontology
☐	☒	Animals and other organisms
☒	☐	Human research participants
☒	☐	Clinical data

Methods

n/a	Involved in the study	
☒	☐	ChIP-seq
☐	☒	Flow cytometry
☒	☐	MRI-based neuroimaging

Antibodies

Antibodies used	All the antibodies used on this study are presented on supplementary table 1, which contains the provide supplier name, catalog number, and clone name.
Validation	On this paper we performed the validation of all the antibodies presented using techniques such as western blot, ELISA, immunofluorescence. We generated knockout cell lines to

Eukaryotic cell lines

Policy information about cell lines

Cell line source(s)	ATCC: Neuro2A (ATCC® CCL-131™); HEK293 (ATCC® CRL-1573); Cho-K1 (ATCC® CCL-61™); ExpiHEK293 (ThermoFisher A14635)
Authentication	Cell line authentication was performed by the provider companies.
Mycoplasma contamination	Cells were negative to mycoplasma contamination
Commonly misidentified lines (See ICLAC register)	N/A

Animals and other organisms

Policy information about studies involving animals; ARRIVE guidelines recommended for reporting animal research

Laboratory animals	Regarding the experiments using mice: Species: C57BL/6; Strain: NCrl; Sex: male; Age: 6-8 weeks.<br>Regarding rabbit antibody development: White New Zealand rabbits, female
Wild animals	The study did not involve wild animals.

Field-collected samples | The study did not involve samples collected from the field.

Ethics oversight | Regarding the experiments using mice: IACUC at Utah State University. Protocol 2775
Regarding rabbit antibody development: IACUC at R.Sargeant Animal Facility operating under NIH OLAW Assurance no, A4182-02

Note that full information on the approval of the study protocol must also be provided in the manuscript.

Flow Cytometry

Plots

Confirm that:

- [x] The axis labels state the marker and fluorochrome used (e.g. CD4-FITC).
- [x] The axis scales are clearly visible. Include numbers along axes only for bottom left plot of group (a 'group' is an analysis of identical markers).
- [x] All plots are contour plots with outliers or pseudocolor plots.
- [x] A numerical value for number of cells or percentage (with statistics) is provided.

Methodology

Sample preparation | One billion yeast cells from each of the constructed libraries were grown in selective medium overnight and then induced for antibody expression and display by switching the cells in induction medium for 24 hours. Next, 100 million of the induced cells were screened with FACS using 1 µM of biotinylated BSA-conjugate and PE-labeled streptavidin. FACS was repeated for an additional 2-4 rounds with progressively lower biotinylated BSA-peptide concentrations to enrich for yeast clones expressing antibody clones that bound the target peptide

Instrument | FACSAria II

Software | FACSDivaV6.1.3

Cell population abundance | The first round of FACS is performed with 100 million cells from the amplified (10-fold) original constructed rabbit antibody library. Sorting in FACS1 using 1 µM FITC-peptide antigen normally results in about 1-5% of the population staining double positive for antibody expression and antigen binding and sorting is continued for 2-4 rounds until >10% double positive clones are observed using 100 nM FITC-labeled peptide antigen.

Gating strategy | An unstained yeast cell sample served to set the PE and FITC negative gates. Double positive cells were gated based on rabbit antibody display detected using PE labeled donkey anti-rabbit IgG from Jackson Immunoresearch (PE fluorescence) and FITC-labeled peptide antigen binding (FITC fluorescence)

- [x] Tick this box to confirm that a figure exemplifying the gating strategy is provided in the Supplementary Information.
